# A Rare Case of Paroxysmal Nocturnal Hemoglobinuria With Bilateral Renal Vein Thrombosis

**DOI:** 10.7759/cureus.8806

**Published:** 2020-06-24

**Authors:** Omair ul haq Lodhi, Shaezal Sohail, Danyal Hassan

**Affiliations:** 1 Internal Medicine, Shifa International Hospital, Islamabad, PAK; 2 Internal Medicine/Nephrology, Shifa International Hospital, Islamabad, PAK

**Keywords:** paroxysmal nocturnal hemoglobinuria (pnh), renal vein thrombosis, cd55 cd59, gpi, piga

## Abstract

Paroxysmal nocturnal hemoglobinuria (PNH) is an acquired hematopoietic stem cell (HSC) disorder characterized by a partial or complete deficiency of glycosyl-phosphatidylinositol (GPI)-linked membrane proteins, which leads to intravascular hemolysis. The loss of CD55 and CD59, two GPI-anchored proteins on red blood cell surfaces, from mutations in the X-linked phosphatidylinositol glycan class A (PIGA) gene, causes unrestricted proliferation of complement activation. The loss of CD59 especially leads to ‘paroxysms’ of acute intravascular hemolysis during events of stress. Extravascular hemolysis also occurs without CD55 as the accumulation of C3 on red blood cell surfaces leads to their destruction by the reticuloendothelial system. Diagnosis of PNH relies primarily on clinical presentation and flow cytometry assays used to detect the GPI-anchored proteins, CD55 and CD59; however, fluorescein‐labeled proaerolysin variant (FLAER) is seen to have a significant advantage over CD55 and CD59. Typical symptoms of the disorder include fatigue, shortness of breath, hemoglobinuria, abdominal pain and bone marrow failure. Thrombosis also occurs secondary to nitric oxide (NO) deficiency, release of procoagulants, increased tissue factor and reduced fibrinolysis. The classification of PNH is subdivided into three types: classical, PNH with another bone marrow disorder and subclinical PNH. Management of hemolysis, thrombosis and pancytopenia is based on the pathogenesis involved. Inhibition of complement in the form of humanized monoclonal antibody against complement C5 (eculizumab) is seen as an emerging treatment option, while stem cell/bone marrow transplant may also be offered. We present a rare case of PNH with bilateral renal vein thrombosis, who was diagnosed with classical PNH on clinical presentation and flow cytometry. He was initially offered bone marrow transplantation but was lost to follow-up and later presented with bilateral renal vein thrombosis. He was managed conservatively with transfusions and anticoagulation, and was discharged for follow-up on an outpatient basis.

## Introduction

Paroxysmal nocturnal hemoglobinuria (PNH) is a rare hematopoietic stem cell (HSC) disorder that results from acquired genetic mutations. PNH typically presents with arterial and venous thrombosis, hemolytic anemia and pancytopenia. The loss of CD55 and CD59, two glycosylphosphatidylinositol (GPI)-anchored proteins on red blood cell surfaces, from mutations in the X-linked phosphatidylinositol glycan class A (PIGA) gene, causes unrestricted proliferation of complement activation resulting in hemolysis [1]. With a prevalence of one to ten in a million population, PNH presents equally in both genders, predominantly in adults, whereas pediatric populations make up 5%-10% of the reported cases [2,3].

The International PNH Registry reported the following clinical findings in 1,610 patients in order of decreasing frequency: fatigue (80%), dyspnea (64%), hemoglobinuria (62%), abdominal pain (44%), bone marrow suppression (44%), erectile dysfunction (38%), chest pain (33%), thrombosis (16%) and renal insufficiency (14%) amongst other symptoms [4].

Data analysis of PNH and thrombosis between 1953 and 2006 retrieved 294 citations. This provided data for 363 cases of PNH with thrombosis, with hepatic vein thrombosis at the highest at 147 (40.7%) and renal vein thrombosis second to last at 12 (3.3%) with a relative risk of 1.79 [5]. Renal manifestations in PNH are not uncommon. A retrospective analysis of 14 patients at a single setup between 1998 and 2004 revealed acute kidney injury (AKI) in six (42.8%), Fanconi syndrome in three (21.4%) and unilateral renal vein thrombosis in two (14.2%) patients [6]. In 2012, a case of PNH with renal vein infarction was also reported [7].

We present a rare case of PNH with bilateral renal vein thrombosis in a 23-year-old gentleman.

## Case presentation

A 23-year-old gentleman, with known case of hepatitis B and PNH, presented to the emergency department with abdominal pain and vomiting. He was previously diagnosed with a loss of fluorescein‐labeled proaerolysin variant (FLAER), CD157 on granulocytes and monocytes (80% loss), and CD59 on erythrocytes (45% loss) on flow cytometry after extensive workup for anemia following a road traffic accident.

Previous workup included a trephine bone marrow biopsy which showed megaloblastic changes in erythroid precursors, a normal red blood cell fragility test, a reticulocyte count of 31%, a lactate dehydrogenase level (LDH) of 3,764 U/L, and a few spherocytes and target cells on peripheral smear.

He had been advised bone marrow transplant, but was lost to follow-up. 

**Figure 1:**
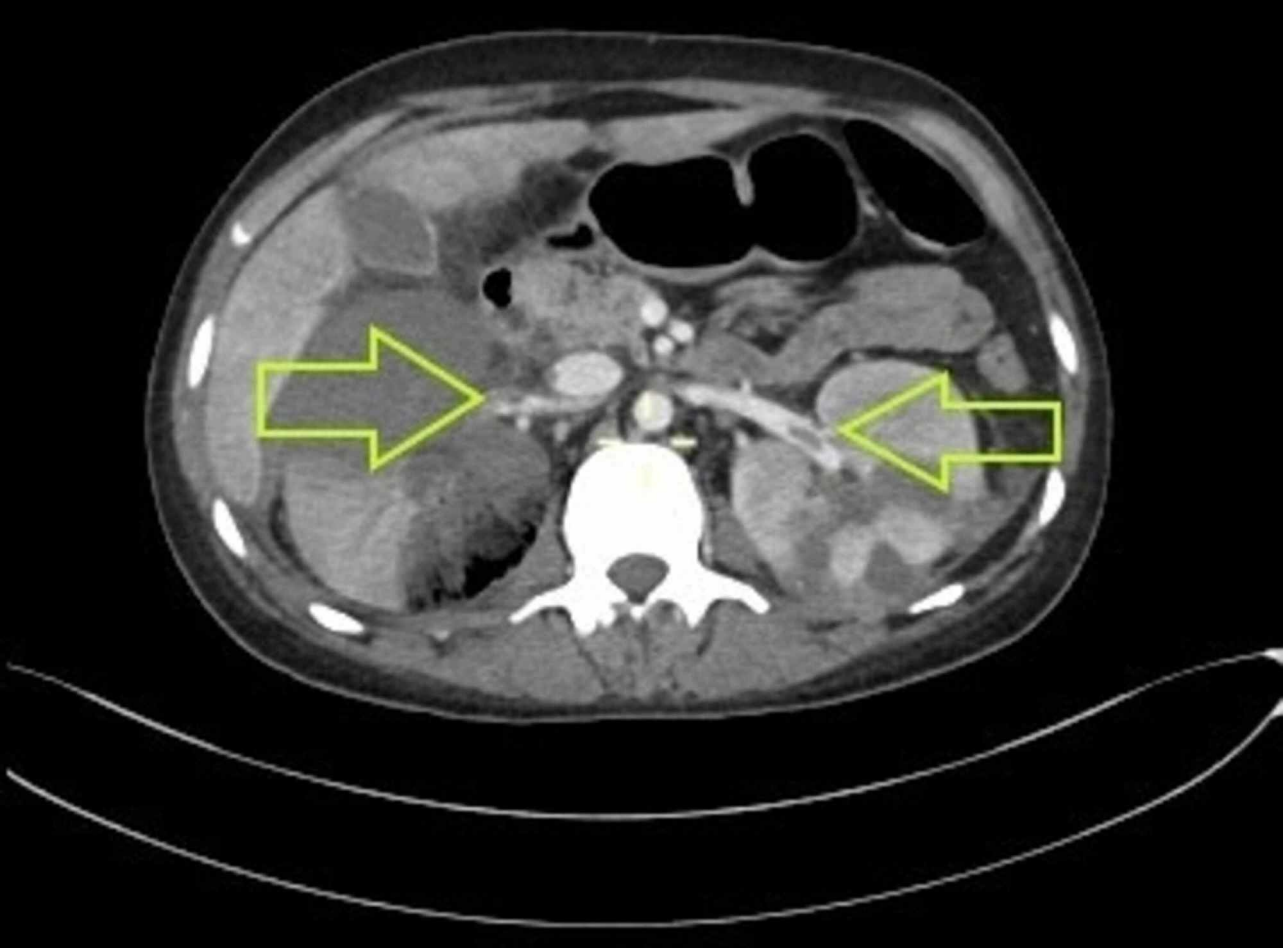
CT with contrast, transverse section showing bilateral renal vein thrombosis Wedge-shaped hypodensities seen in bilateral kidneys, more in the right kidney which is swollen and enlarged. Filling defect noted involving the right renal vein at its ostium and extending into the hilar region. At the same level, there is extension into the inferior vena cava as well. Similar filling defect is noted in the left renal vein at the hilum. Both filling defects are shown by the yellow arrows in the image.

**Figure 2:**
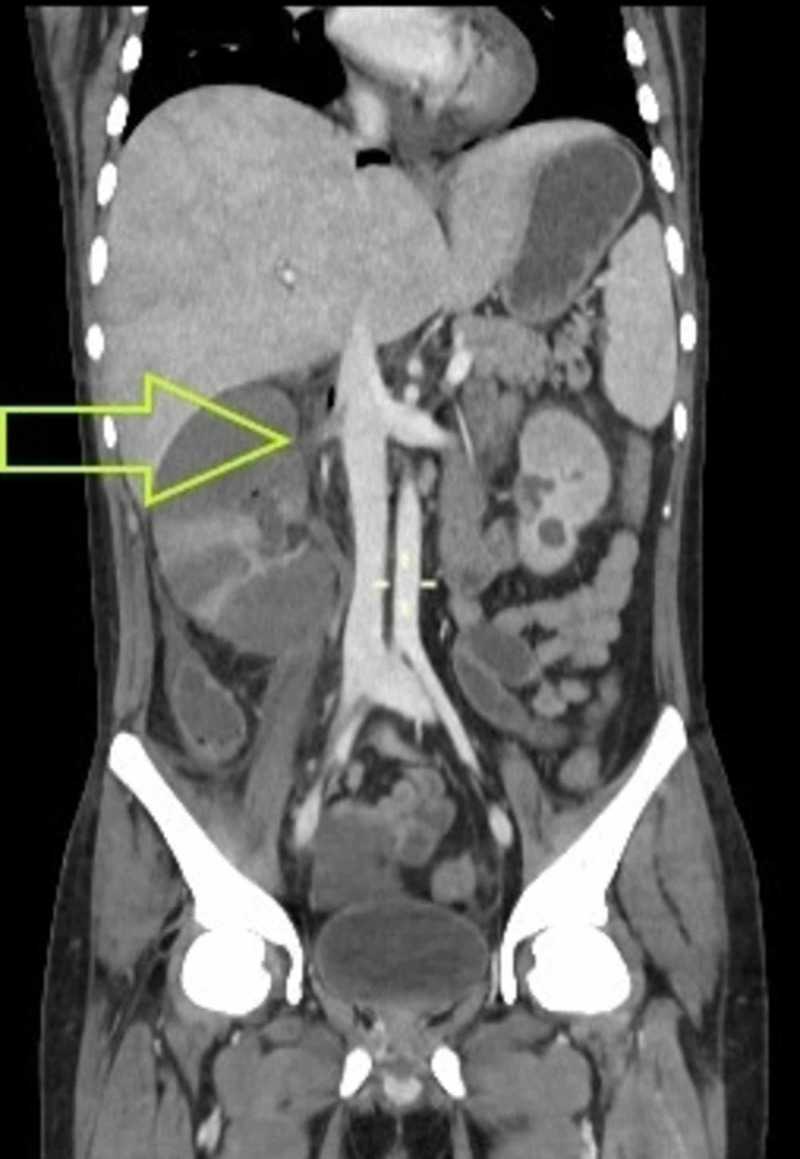
CT with contrast, axial section showing right renal vein thrombosis extending into the inferior vena cava Filling defect is noted involving the right renal vein at it ostium and is seen extending into the hilar region. At the same level, it is seen extending into the inferior vena cava as well. Filling defect is shown by the yellow arrow.

Now, on this presentation, he had marked pallor, scleral icterus and a mildly tender abdomen. Workup showed a low hemoglobin and platelet count, AKI and hypokalemia. A CT scan of the abdomen and pelvis with contrast was done, which revealed splenic and bilateral renal vein thrombosis, hepatomegaly with thrombosis of right hepatic vein, middle hepatic vein and anterior division of right portal vein, mild abdominopelvic ascites, mild pericardial effusion and bilateral lobar nephronia. The bilateral renal vein thrombosis can be seen in Figures 1, 2.

Hospital course and management

The patient was admitted and managed on the lines of bicytopenia, AKI, extensive venous thrombosis, renal abscess and PNH. A multidisciplinary approach was adopted with hematology, nephrology, urology, interventional radiology and gastroenterology on board. He received both platelet and packed red blood cell transfusions, intravenous hydration and intravenous antibiotics. He underwent abscess drainage and bilateral double J (DJ) stenting. Anticoagulation was not offered initially due to a low platelet count, but heparin was started eventually. Additionally, eculizumab was unavailable.

His hospital stay lasted a total of seven days. Hematological and biochemical trends can be seen in Tables 1, 2. Clinically, he displayed a marked improvement following abscess drainage and bilateral DJ stent placement. Blood and body fluid cultures revealed Escherichia coli, for which he received intravenous meropenem and ertapenem. At the time of discharge, his creatinine had dropped from an initial value of 5.3 mg/dL to a static 2.7-2.8 mg/dL, hypokalemia had resolved, blood counts had improved and he was afebrile, tolerating orally and mobilized. He was advised to follow up as an outpatient to discuss further treatment options.

**Table 1:** Serial Laboratory Investigations NP: not performed, INR: international normalized ratio

Laboratory Investigations	Day 1	Day 2	Day 3	Day 4	Day 5	Day 6	Day 7	Normal Values
Serum sodium	133	NP	134	134	133	133	132	(136-144 mEq/L)
Serum potassium	2.8	NP	3.2	2.9	3.5	3.9	3.2	(3.7-5.2 mEq/L)
Serum chloride	100	NP	99	98	101	102	NP	(101-111 mEq/L)
Serum bicarbonate	21	NP	16	13	13	13	15	(22-28 mEq/L)
Serum creatinine	5.3	NP	4.33	3.96	3.51	3.19	2.81	(0.8-1.2 mg/dL)
Serum urea	65	NP	71	68	63	64	56	(7-20 mg/dL)
Alanine aminotransferase	NP	29	NP	17	14	12	13	(7-56 U/L)
Aspartate aminotransferase	NP	22	NP	NP	NP	34	40	(10-40 U/L)
Alkaline phosphatase	NP	247	NP	NP	NP	344	330	(44-147 U/L)
Gamma-glutamyl transferase	NP	60	NP	NP	NP	172	156	(9-48 U/L)
Total bilirubin	NP	5.35	NP	15.28	20.12	19.9	21.53	(0.3-1.9 mg/dL)
Direct bilirubin	NP	4.12	NP	NP	NP	13.16	18.87	(0-0.3 mg/dL)
Hemoglobin	6.9	6.4	6.7	7.8	8	7.9	8.2	(12-5.5 g/dL)
White blood cell, total	6,310	15,060	11,600	16,000	19,240	17,780	14,590	(4,500-11,000/μL)
Platelet count	91,000	16,000	14,000	24,000	17,000	22,000	32,000	(150,000-400,000/μL)
C-reactive protein	NP	382.34	NP	NP	311.49	NP	311	(0-3.0 mg/L)
Prothrombin time/INR	NP	1.3	NP	1.27	1.22	NP	1.2	(10-13.5 seconds/0.90-1.15)
Lactate dehydrogenase	NP	1046	NP	NP	3764	NP	NP	(140-280 U/L)
Reticulocyte count	NP	31%	NP	NP	NP	NP	NP	(0.5%-2.5% cells)

**Table 2:** Serum Antibodies and Cultures FLAER: fluorescein‐labeled proaerolysin variant, E. coli: Escherichia coli

Laboratory Investigation	CD59	CD157	CD55	FLAER	Blood Culture	Urine Culture	Body Fluid Culture	CD15, CD45, CD64, CD235a
Results	Detected	Detected	Not performed	Detected	E. coli	No growth	E. coli	Not detected

## Discussion

The International PNH Registry documented the symptoms of fatigue, shortness of breath, hemoglobinuria, abdominal pain and bone marrow suppression as being the most frequent symptoms of PNH [4]. The first two can be attributed to anemia, which in the case of PNH is multifactorial with both hemolysis and impaired erythropoiesis involved. A complete blood count is useful in that both thrombocytopenia and leukopenia suggest a stem cell dysfunction, while reticulocyte count is a good indicator of the marrow response. LDH is a useful marker of hemolysis, while urine hemosiderin also suggests chronic intravascular hemolysis. Renal dysfunction (low erythropoietin) and iron deficiency (hemosiderinuria/hemoglobinuria) may contribute to anemia in PNH as well [8].

Less commonly, patients with PNH can also present with manifestations of smooth muscle dystonia such as abdominal pain, dysphagia and erectile dysfunction and are at a six times increased risk of chronic kidney disease, with multiple mechanisms, including tubular atrophy, interstitial fibrosis and microinfarcts playing a role [1,6]. Our patient presented with abdominal pain, which could, however, also be attributed to extensive intra-abdominal thrombosis. At the time of initial presentation in 2018, our patient’s renal function was seen to be largely preserved with normal serum creatinine levels and imaging. During his current admission, however, he was found to have deteriorated with chronic changes evident even after the AKI had resolved.

A definite diagnosis of PNH requires demonstration of deficiency of two or more GPI-anchored proteins detected by flow cytometry and also FLAER. However, it must be kept in mind that 60% of patients with acquired aplastic anemia and 20% of patients with myelodysplastic may have GPI-anchored protein-deficient cells [8,9]. Other markers that may help in diagnosis include CD16, CD24, CD66b, CD48 and CD157. FLAER has a compelling advantage over CD55 and CD59 as the latter works less efficiently in high sensitivity analysis. FLAER binds specifically to GPI-anchored proteins as its binding is less sensitive to maturational stage of cells and can also be used in multicolor combinations with non-GPI antigens and monoclonal antibodies to GPI linked in order to detect PNH clones [10]. Our patient was diagnosed with a loss of FLAER, CD157 on granulocytes and monocytes (80% loss), and CD59 on erythrocytes (45% loss) on flow cytometry. Our patient also underwent a trephine bone marrow biopsy which showed megaloblastic changes in erythroid precursors and a normal red blood cell fragility test. Repeat flow cytometry was not performed since no definitive treatment was received. 

The classification of PNH is subdivided into three types to include the deviations in clinical presentation and history. Classical PNH with clinical evidence of intravascular hemolysis (raised serum LDH and indirect bilirubin, and abnormally low concentration of serum haptoglobin) is often found to have greater than 50% of PNH granulocytes. The second type is PNH with another bone marrow disorder, such as aplastic anemia, myelodysplastic syndrome (MDS) or other myelopathy, and the third type is subclinical PNH with no clinical or laboratory hemolysis [8]. Our patient, at the time of diagnosis of PNH, presented with fatigue, shortness of breath and recalled multiple episodes of ‘dark-colored urine’ when febrile or under stress. His workup also revealed involvement of 80% granulocytes with elevated reticulocytes and LDH levels, thus fitting in with the picture of classic PNH. Our patient, as common in classic PNH, also went on to develop extensive thrombosis.

Thrombosis is the leading cause of mortality in PNH, with intra-abdominal (hepatic) and cerebral veins being the most common sites [1]. Thrombosis occurs secondary to nitric oxide deficiency, release of procoagulants, increased tissue factor and reduced fibrinolysis [9]. Our patient did have extensive abdominal thrombosis (hepatic, portal and splenic veins) but also had bilateral renal vein thrombosis, a relatively less common presentation. The role of prophylactic therapy is still up to debate, but the fairly high risk of thromboembolic complications have led some authors to speak in favor of it. The study reported no thromboembolic events in the 39 patients on warfarin prophylaxis as compared to a 36.5% 10-year risk of thrombosis in the 56 patients who did not receive warfarin [11]. For acute thromboembolic events, heparin is the mainstay of treatment with radiologic intervention as another option [8]. Our patient was initially not offered anticoagulation due to a markedly low platelet count but was later started on heparin after multiple platelet transfusions.

In patients with clinical evidence of hemolysis (classic PNH and PNH/aplastic anemia), after diagnosis, flow cytometry analysis of both granulocytes and erythrocytes is recommended biannually for two years and then annually unless there is evidence of clinical progression. In patients without clinical evidence of hemolysis with aplastic anemia or refractory anemia-MDS, annual flow cytometry is recommended. Bone marrow analysis is not recommended for routine diagnosis of PNH, although GPI-anchored proteins can be detected on CD34 bone marrow cells using two-color flow cytometry. PIGA mutations, although confirm the diagnosis of PNH, are only limited for research purposes due to technical challenges [8].

Moving on to management, the hemolysis in PNH is associated with complement-mediated cytolysis; hence, inhibition of complement in the form of humanized monoclonal antibody against complement C5 (eculizumab) is seen as an emerging treatment option. It is the only US Food and Drug Administration approved therapy for PNH and is given intravenously every seven days for the first five weeks and then twice a week subsequently. The drug was found to be effective against intravascular hemolysis, decreasing the need for transfusions as well as reducing the risk of thrombosis, and it also showed a positive impact on renal function. However, there is insufficient data as yet on the long-term outcomes [1,9]. Additionally, the drug is expensive and must be given indefinitely for a sufficient response. These factors, and the drug's non-availability, rendered it an unsuitable treatment option for our patient.

Regarding bone marrow/stem cell transplantation, debates are still ongoing. While it is the only curative treatment for PNH, the transplant-associated risks and complications are ever present. A study conducted by the French Society of Hematology on a group of 220 patients with PNH revealed a median survival rate of 12 years, with some risk factors associated with worse outcomes [12]. Factors such as occurrence of thrombosis, thrombocytopenia at the time of diagnosis, progression to pancytopenia and transformation to MDS or acute leukemia might influence the decision regarding transplant. In countries where eculizumab is unavailable, a more liberal approach may be sought. Our patient was advised bone marrow transplant but was lost to follow-up.

## Conclusions

We conclude that clinicians should be aware of rare complications and their mechanisms so that a timely diagnosis can be made and prompt treatment can be initiated. Renal vein thrombosis is uncommon in patients with PNH, and hence may be missed. In our lost to follow-up case, prompt diagnostic evaluation revealed multisystemic thrombosis including bilateral renal vein thrombosis, and he was managed accordingly. Further reporting of such cases needs to be done to strengthen the association, so that an accurate diagnosis can be made and benefit the patient as much as possible.
